# Beverage Availability and Price: Variations by Neighborhood Poverty Level in New York City

**DOI:** 10.1089/heq.2021.0069

**Published:** 2022-04-27

**Authors:** Marie A. Bragg, Pasquale E. Rummo, Tenay Greene, Josh Arshonsky, Amaka V. Anekwe, Tamar Adjoian Mezzacca, Shannon M. Farley

**Affiliations:** ^1^ Department of Population Health, New York University School of Medicine, New York, New York, USA.; ^2^ New York University College of Global Public Health, New York, New York, USA.; ^3^ New York City Department of Health and Mental Hygiene, Bureau of Chronic Disease Prevention and Tobacco Control, Long Island City, New York, USA.; ^4^ New York, NY.; ^5^ ICAP, Columbia University, Columbia University Mailman School of Public Health, New York, NY USA.

**Keywords:** obesity, food environment, beverages, sugary drinks

## Abstract

**Objective:**

To describe the variability in the availability and price of sugary drinks, low-calorie drinks, and water/seltzer across high- and low-poverty census tracts in the five boroughs of New York City (NYC).

**Design:**

Cross-sectional study. Our primary analysis compared the overall sample of beverages. Secondary analyses included tests for differences in the availability of beverage categories by neighborhood poverty level.

**Setting:**

We collected data from 106 stores (31 supermarkets, 29 convenience stores, 29 pharmacies, 9 Targets, and 8 Dollar Trees) in NYC. Fifty-four stores were located in high-poverty census tracts and 52 were located in low-poverty census tracts.

**Results:**

The mean Price per 0.03-liter of sugary drinks across the sample was $0.08, which was significantly higher than the price of low-calorie drinks ($0.07, *p*=0.01) but not different from water/seltzer ($0.08, *p*=0.65). Sugary drinks and water/seltzer were available in 91% of retailers, and low-calorie drinks were available in 87% of retailers. There was no statistical difference in availability of sugary drinks compared with low-calorie drinks or water/seltzer overall or within high- or low-poverty census tracts. Analyzed by store type, the mean price per ounce of sugary drinks differed significantly from water/seltzer at convenience stores, pharmacies, and Target stores (bodegas: $0.08 vs. $0.09, *p*=0.03; pharmacies: $0.11 vs. $0.08, *p*=0.02; Target stores: $0.07 vs. $0.09, *p*=0.01).

**Conclusions:**

Sugary drinks were available in most food retail settings in NYC, with little variation by census tract poverty level. Interventions that raise the price of sugary drinks to make healthier alternatives, such as water, the more affordable option should be considered.

## Introduction

Sugary drinks are high in calories but have little or no nutritional value, and they represent the largest source of added sugars in the diet of Americans of ages 2 years and older.^1^ U.S. federal guidelines state that added sugar should account for <10% of daily calories; for example, in a 2,000 calorie diet, that would equate to <200 calories.^2^ Yet a single serving of soda may exceed the daily recommendation for many people. Sugary drinks are linked to weight gain; other associated negative health outcomes include heart disease, type-2 diabetes, and cavities.^3–10^ Consumption of sugary drinks is both a public health issue and a health equity concern.

Specifically, beverage companies spend hundreds of millions of dollars on sugary drink promotion^11^ and heavily market sugary drinks to low-income communities^12^ and communities of color.^13^ One study found 4.35 higher odds of in-store sugary drink marketing during Supplemental Nutrition Assistance Program (SNAP) benefit issuance days—the first 9 days of the month—compared with other days of the month in census tracts with high percentages of residents who use SNAP.^12^ These factors, which occur alongside policies and practices based on a history of racism and discrimination in the United States,^14^ may contribute to higher rates of sugary drink consumption and inequities in rates of diet-related diseases based on income and race.^15–20^

About 70% of added sugars consumed in the United States are purchased in retail establishments such as supermarkets and convenience stores—compared with 16% in restaurant settings^21^—this is indicative of the key role of retail settings in the consumption of sugary drinks.^2^ Retail settings influence consumer shopping behavior through practices such as product availability and pricing. These practices, among others, comprise “commercial determinants of health,” defined as “strategies and approaches used by the private sector to promote products and choices that are detrimental to health.”^22^

This study is the first to assess beverage availability and pricing in a variety of New York City (NYC) retail environments across all five boroughs, and to examine differences by census tract poverty level. Quantified information about the retail beverage landscape may heighten understanding of how to better influence health-promoting consumer behavior and inform public health strategies to address the overconsumption of sugary drinks.

## Methods

### Sample

We randomly selected 1 low- and 1 high-poverty census tract in each of the 5 boroughs of NYC, for a total of 10 tracts. In accordance with NYC Department of Health and Mental Hygiene (Health Department) guidance, low-poverty (i.e., higher income) tracts were defined as areas where <10% of the population had an income level <100% of the Federal Poverty Level (FPL), and high-poverty (i.e., lower income) tracts were defined as areas where at least 20% of the population had an income level <100% of the FPL (based on U.S. Census Bureau data).^23^

Next, we used data from the New York State (NYS) Department of Agriculture and Markets to randomly select 11 chain and independent food retail outlets in each of the 10 census tracts, for a total of 110 stores. In each tract, three of each of the following outlets were selected: supermarkets (defined as a food retail outlet with >929.03 square meters or a store name that represented a chain supermarket [e.g., Key Foods and Whole Foods]); pharmacies (i.e., one CVS [retail pharmacy chain] and two local/independent pharmacies); and convenience stores (i.e., one 7-Eleven store and two local/independent corner stores, defined as <371.61 square meters).

**FIG. 1.**
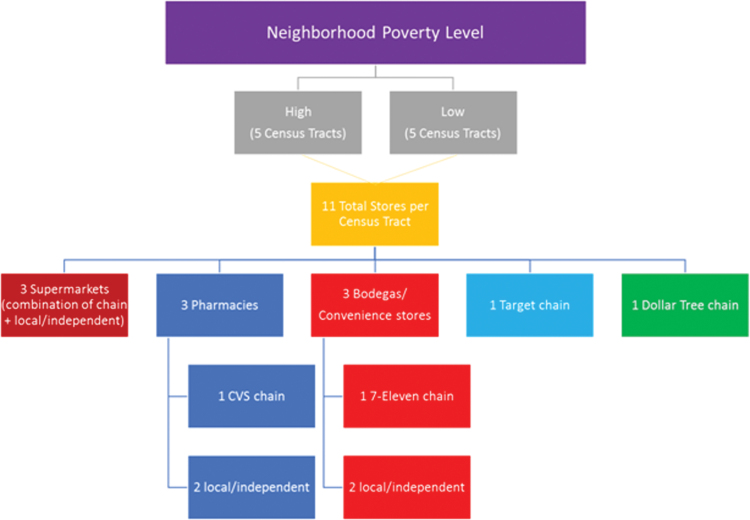
Stores included in sample, beverage pricing, and availability study, NYC 2017. NYC, New York City.

These outlet types were included because they are among the most common purchase locations of sugary drinks in NYC.^24,25^ The remaining 2 outlet types included chain retailers found in each of the 5 boroughs: 10 Target retail stores and 10 Dollar Tree stores, 1 each in the census tract closest to the randomly selected tracts. These two chains were selected because they are commonly found within all five NYC boroughs (Fig. 1).

### Product definitions

For the purposes of this study, five main categories of beverages were defined: sugary drinks, low-calorie drinks, water/seltzer, plain/unsweetened milk, and 100% juice. The analyses presented here focus on sugary drinks, low-calorie drinks, and water/seltzer only. Sugary drinks were broadly defined as products with added caloric sweetener and 24 or more calories per 0.01-liter, which is consistent with how such beverages were defined in a previously proposed sugary drink regulation in NYC.^26^ Because there is a wide variety of drink products available in the United States, sugary drinks were further subcategorized into the following groups: carbonated soft drinks (i.e., soda), sweetened iced tea, fruit drinks/vitamin-enhanced waters, sports drinks, and energy drinks.

Low-calorie drinks served as a counterpart to sugary drinks and had low- or no-calorie sweetener and <24 calories per 0.01-liter. Water/seltzer included unsweetened bottled or canned products that could be either plain or flavored. We grouped seltzer and water for the following reasons. Although seltzer can be used as a mixer for hard liquor, it has grown in popularity as a standalone drink over the years, with sales of sparkling mineral water projected to reach $6 billion this year.^27^ Many people report drinking seltzers to complement their low calories or to substitute water when needing hydration.^28^

### Measurement tool

The Nutrition Environment Measures Survey for Beverages (NEMS-B) was adapted for use in food retail outlets in this study. NEMS-B was based on similar tools (e.g., NEMS in stores) that are validated observational measures of food retail environments.^29,30^ It is designed to evaluate the availability, price, and promotion of different beverage types in food retail stores. We modified the NEMS-B to include beverage choices in groups as already defined. Eighteen data collectors completed the online NEMS training tool provided by researchers at the University of Pennsylvania.

Based on the Rudd Center's 2011 Sugary Drinks FACTS Report,^31^ the top two to three highest selling sugary drink brands from each group were selected as target products, as long as the company also sold a low-calorie drink counterpart (e.g., Coke and low-calorie Coke). The sugary drink category included the following subcategories and brands: (1) carbonated soft drinks: Coke and Pepsi; (2) sweetened iced tea: Snapple and AriZona; (3) energy drinks: Monster and Red Bull; (4) sports drinks: Gatorade and Powerade; and (5) fruit drinks/vitamin-enhanced waters: Fuze, V8 Splash, and Vitamin Water. The low-calorie drinks category included the counterparts to each sugary drink brand.

The water/seltzer category included Aquafina for the plain water brand and Poland Spring (or any generic brand, if Poland Spring was unavailable) for the plain or flavored seltzer brand. A generic or store brand was also included for each beverage category as a mechanism to ensure the sample reflected the true range of pricing among drink categories. For comparability, data collectors prioritized collecting price and availability data for 0.35-liter and 0.59-liter beverage sizes where possible. For brands that did not commonly have 0.35-liter and 0.59-liter size options (e.g., energy drinks, V8), data collectors recorded the price and size of other common small and large size options for that brand (e.g., 0.01-liter and 0.47-liter). In addition, the same brands within each beverage group were used for data collection.

### Data collection procedures

After completing the NEMS training, data collectors conducted a pilot test of the survey instrument at five stores in a census tract that was not part of the final sample and completed the modified NEMS-B on tablets. To ensure high-quality data collection across all retail settings, inter-rater reliability was established using this pilot sample of five stores. All raters reached a Krippendorf's alpha level of 0.7 or higher. After establishing reliability of the pilot sample, the stores were divided among the 18 data collectors. For convenience purposes, data collectors were assigned stores that were geographically clustered together. Each store visit lasted ∼1 to 1.5 h. Institutional review board approval was not required, because the study did not involve human participants.

Availability was assessed by noting which of our target products were sold at a given retailer (yes/no). Price was assessed by recording the posted price of target products within stores. The prices of refrigerated beverages were prioritized, but if these were unavailable, prices of unrefrigerated beverages were used instead. When the price was not posted, store personnel were asked the price of each product. After pricing data were collected, NYS sales tax of 8.875% was added to recorded prices of eligible beverages (i.e., sugary drinks, low-calorie drinks, and bottled water/seltzer) at stores that utilized barcode scanners.^32^

A $0.05 bottle/can deposit was also added to eligible beverages sold at stores with scanners.^33^ Eligible beverages were those sold in sealed glass, metal, and plastic containers smaller than 1 gallon or 3.78 liters. Sales tax and bottle/can deposit were not added to the prices of beverages sold at stores without UPC scanners (i.e., small, independent stores), since these additional costs are typically built into the posted price and are not added at checkout.

### Outcomes

Data collection provided information on a variety of available products, the distribution of which was not necessarily reflective of local purchase and consumption patterns. For example, energy drinks generally cost more per ounce than other types of sugary drinks but are purchased in much smaller volumes. To adjust for this, sugary and low-calorie drink subcategories were weighted by the relative proportion of volume sold in the NYC retail environment using Nielsen sales data from 2015 (sugary drinks: 48.5% soda, 14.6% sweetened iced tea, 22.0% fruit drinks/vitamin-enhanced waters, 13.2% sports drinks, 1.7% energy drinks; low-calorie drinks: 70.5% soda, 12.9% sweetened iced tea, 3.1% fruit drinks/vitamin-enhanced waters, 8.7% sports drinks, 4.7% energy drinks).

This strategy is consistent with another recent study of beverage pricing.^34^ After weighting, subcategories of beverages were collapsed into three main categories for analyses of availability and price: sugary drinks, low-calorie drinks, and water/seltzer. Price per ounce of 0.35-liter and 0.59-liter drinks of the same beverage category within stores was compared using paired *t*-tests. No significant differences were found; therefore, both sizes were included in the primary analyses.

### Statistical analyses

Availability of beverage categories and subcategories (yes/no) was summarized with frequency counts and percentages. McNemar's test for matched paired data was used to test for differences in the availability of sugary drinks relative to low-calorie drinks and to water/seltzer in the overall sample; and a chi-squared test was to test for differences in the availability of beverage categories by neighborhood poverty level. Pricing data are summarized using means, standard deviations, and ranges. To standardize across potentially varying product sizes, price was analyzed on a per-ounce basis.

Paired *t*-tests were used to test for differences in the mean price of sugary drinks relative to low-calorie drinks and to water/seltzer in the overall sample, and independent samples *t*-test were used to test for differences in the availability and price of beverage categories by neighborhood poverty level. We also used paired *t*-tests to test for differences in the mean price of sugary drinks, low-calorie drinks, and water/seltzer within store types, but lacked sufficient variation to estimate differences in presence within store types. All analyses were performed using Stata version 15.0 (StataCorp LP, College Station, TX).

## Results

### Retail sample

The final sample included 106 of the initially sampled 110 stores (31 supermarkets, 29 convenience stores, 29 pharmacies, 9 Targets, and 8 Dollar Stores), of which 54 were in high-poverty census tracts and 52 were in low-poverty census tracts. Four stores were excluded, because store staff asked the researchers to leave the premises, resulting in a 96% completion rate.

### Availability

There were no significant differences in availability of sugary drinks compared with either low-calorie drinks or water/seltzer overall (Table 1). We also observed no differences in the presence of sugary drinks (*p*=0.47) or low-calorie drinks (*p*=0.0) by census tract poverty level. The presence of water/seltzer, however, was significantly lower in high-poverty census tracts (84.6%) versus low-poverty census tracts (96.3%) (*p*=0.04). In all census tracts combined, sugary drinks and water/seltzer were available in 91% of retailers, and low-calorie drinks were available at 87% of retailers. In high-poverty neighborhoods, all three beverage categories were available at >80% of retailers (sugary drinks: 89%; low-calorie drinks: 83%; water/seltzer: 85%).

**Table 1.** Differences in Presence and Price Per Ounce Between Sugary/Low-Calorie Drinks and Water/Seltzer, Overall and by Neighborhood Poverty Level

	Presence (*n*)	Presence (%)	*p*	Price per oz (mean)	Price per oz (SD)	*p*
All CTs (*n*=106)						
Sugary drinks	96	90.6	—	0.08	0.03	—
Low-calorie drinks	92	86.8	0.05^a^	0.07	0.02	0.01^b^
Water and seltzer	96	90.6	n/a^c^	0.08	0.05	0.65^b^
High-poverty CTs (*n*=52)						
Sugary drinks	46	88.5	—	0.08	0.04	—
Low-calorie drinks	43	82.7	—	0.07	0.02	—
Water and seltzer	44	84.6	—	0.08	0.06	—
Low-poverty CTs (*n*=54)						
Sugary drinks	50	92.6	0.47^d^	0.08	0.03	0.37^e^
Low-calorie drinks	49	90.7	0.22^d^	0.07	0.02	0.19^e^
Water and seltzer	52	96.3	0.04^d^	0.08	0.02	0.59^e^

The presence (%) reflects the percentage among stores with nonmissing data. ^a^*p*-value reflects the results of a McNemar's chi square test for comparing the presence of sugary drinks with low-calorie drinks and water and seltzer (separately).

^b^ *p*-value reflects the results of a paired *t*-test for comparing the price per oz. of sugary drinks with low-calorie drinks and water and seltzer (separately).

^c^ n/a indicates that we lacked sufficient variation to estimate differences between measures.

^d^ *p*-value reflects the results of a chi-squared test for comparing the availability of sugary drinks, low-calorie drinks, and water and seltzer (separately) between high-poverty and low-poverty census tracts.

^e^ *p*-value reflects the results of an independent samples *t*-test for comparing the price per oz. of sugary drinks, low-calorie drinks, and water and seltzer (separately) between high-poverty and low-poverty census tracts.

CT, census tract; SD, standard deviation.

In low-poverty areas, all three categories were available at >90% of retailers (sugary drinks: 93%; low-calorie drinks: 91%; water/seltzer: 96%). When analyzed by store type, no differences existed in the availability of sugary drinks compared with low-calorie drinks or water/seltzer (Table 2).

**Table 2.** Differences in Presence and Price Per Ounce Between Sugary/Low-Calorie Drinks and Water/Seltzer, Overall and by Store Type

	Presence (*n*)	Presence (%)	price per 0.03-liter (mean)	price per 0.03-liter (SD)	*p* ^a^
All store types (*n*=106)					
Sugary drinks	96	90.6	0.08	0.03	—
Low-calorie drinks	92	86.8	0.07	0.02	0.01
Water and seltzer	96	90.6	0.08	0.05	0.65
Dollar Tree (*n*=8)					
Sugary drinks	8	100.0	0.05	0.02	—
Low-calorie drinks	8	100.0	0.06	0.02	0.07
Water and seltzer	8	100.0	0.06	0.02	0.16
Convenience stores (*n*=29)					
Sugary drinks	28	96.6	0.08	0.01	—
Low-calorie drinks	28	96.6	0.07	0.02	0.16
Water and seltzer	29	100.0	0.09	0.01	0.03
Supermarkets (*n*=31)					
Sugary drinks	31	100.0	0.08	0.02	—
Low-calorie drinks	28	90.3	0.07	0.02	0.03
Water and seltzer	30	96.8	0.07	0.08	0.91
Pharmacy (*n*=29)					
Sugary drinks	20	69.0	0.11	0.05	—
Low-calorie drinks	19	65.5	0.08	0.03	0.02
Water and seltzer	20	69.0	0.08	0.01	0.02
Target (*n*=9)					
Sugary drinks	9	100.0	0.07	0.01	—
Low-calorie drinks	9	100.0	0.08	0.01	0.65
Water and seltzer	9	100.0	0.09	0.01	0.01

The presence (%) reflects the percentage among stores with nonmissing data. We lacked sufficient variation to estimate differences in presence within store types.

^a^ *p*-value reflects the results of a paired *t*-test for comparing the price per oz. of sugary drinks with that of low-calorie drinks and water and seltzer (separately).

### Pricing

The mean price per 0.03-liter of sugary drinks across the sample was $0.08, which was similar to the price per ounce of water/seltzer ($0.08, *p*=0.65) and significantly more expensive than the mean price of low-calorie drinks ($0.07, *p*=0.01) (Table 1). There were no differences in price by neighborhood poverty level for sugary drinks ($0.08 vs. $0.08, *p*=0.37), low-calorie drinks ($0.07 vs. $0.07, *p*=0.19), or water/seltzer ($0.08 vs. $0.08, *p*=0.59).

The mean price per 0.03-liter of sugary drinks versus low-calorie drinks did not significantly differ at Dollar Tree stores, convenience stores, or Target stores (Table 2). At supermarkets and pharmacies, low-calorie drinks were significantly less expensive per ounce than sugary drinks ($0.07 vs. $0.08, *p*=0.03; $0.08 vs. $0.11, *p*=0.02, respectively) (Table 2). The mean price per ounce of sugary drinks versus water/seltzer differed significantly at convenience stores, pharmacies, and Target stores (convenience stores: $0.08 vs. $0.09, *p*=0.03; pharmacies: $0.11 vs. $0.08, *p*=0.02; Target stores: $0.07 vs. $0.09, *p*=0.01).

## Discussion

Our findings show that sugary drinks are pervasive in the NYC retail environment. Nearly all store locations in our sample sold sugary drinks; their ubiquity at pharmacies is especially concerning, given the ostensible purpose of these retailers is one of health promotion. The primary finding from our study is that the availability of water/seltzer is significantly lower in high-poverty areas than in low-poverty areas. Even though the price of beverages is similar, the lower availability of water in high-poverty areas is concerning because equivalent pricing of sugary drinks and water/seltzer may not provide a strong enough incentive toward selecting water or seltzer, as consumers' decision making is impacted by many factors beyond price.^24^

Food and beverage companies tend to employ marketing strategies that capitalize on place, price, product, and promotion—known as the 4 P's of foundational marketing. To drive purchasing and ultimately increase sales, companies use place-based marketing, including offline and online promotion tactics; sell their products for low prices; and develop unique products that appeal to particular groups of consumers.^35^

For example, a recent study found that outdoor sugary drink advertising in NYC is prevalent in retail-dense areas, with a higher density of sugary drink ads observed in neighborhoods with a greater percentage of residents who are Black, as well as with higher poverty, and lower education levels.^36^ If water/seltzer is less available and sugary drink ads are prevalent in high-poverty areas compared with low-poverty areas, residents may be persuaded to purchase sugary drinks, especially if the price is the same.

Low-calorie drinks were priced similarly to sugary drinks in both high-poverty and low-poverty census tracts. Compared with sugary drinks, low-calorie drinks do not have as robust an evidence base regarding long-term impacts on health. As such, public health experts acknowledge further research is needed to assess impacts related to low- and no-calorie sweetener consumption, particularly among children.^37^ Given the lack of consensus on the health impacts of such beverages, pricing schemes that might incentivize consumption of low-calorie drinks over water, which is known to be the healthiest choice, may be seen as problematic.

Our findings support those of Leider and Powell,^34^ who found that sugary drink prices did not vary by income or neighborhood poverty level. However, our findings differed in that the prices of sugary drinks and water/seltzer were similar in most retail outlets in our sample, whereas Lieder and Powell found that water was significantly less expensive than sugary drinks at food stores in four U.S. metro areas. This may be due to potential differences in the price elasticity of bottled water in these metro areas compared with NYC, possibly driven by differences in perceptions of the safety of consuming tap water.^38^

In our study, Dollar Tree stores sold sugary drinks for only five cents/ounce, offering very-low-cost access to these products. Put in perspective, this equates to just $0.60 for a 0.35-liter can or $1.00 for a 0.59-liter bottle. Although we included a small sample of Dollar Trees (*n*=8), dollar stores in general have recently gained media attention for serving as the primary food source for many low-income families and predominantly supplying low-quality nutrient-poor highly processed foods.^39–41^

Not only do these retailers provide access to inexpensive unhealthy products, their presence may also displace stores with healthier, although market rate, food options.^42^ Broader policy changes (e.g., subsidizing healthier foods and beverages for people who receive SNAP benefits) are urgently needed to enable families with lower incomes to afford healthy products.

Policy approaches that incentivize price-sensitive consumers to select the healthier of the two beverage options (i.e., water/seltzer in lieu of sugary drinks) would be preferable to one where both options are equal. Strategies to increase the relative price of sugary drinks may be particularly effective; for instance, one study showed that a 10% increase in the price of sugary drinks would lead to a 12% decrease in consumption.^43^ A growing body of empirical evidence suggests beverage taxes reduce sugary drink purchases and intake and can increase water consumption,^44–49^ including among consumers with lower income.^50^

Some price-based policy approaches to reduce sugary drink consumption, including minimum pricing and discount bans, find their roots in the tobacco control policy approaches. Requiring sugary drink retailers to have a license is another public health strategy that may help regulate practices in these spaces.^51^ These approaches are as of yet untested for sugary drinks but have been identified by public health experts as innovative policies warranting consideration.^51^

This study had several limitations to note. First, the cross-sectional nature of the survey only allows for presenting data at a single time point. Second, data on availability and prices of specific beverages were taken from only a sample of retailers that do not represent the full scope of where beverages are sold, and a limited number of specific chains (e.g., CVS, 7-Eleven) were included. Third, not all retailers were located precisely within sampled census tracts; in these instances, stores in nearby census tracts were used instead.

## Conclusions

Store environments may play a critical role in short-term consumer purchasing and consumption patterns, which may contribute to longer term negative low-calorie–related health outcomes. The lower availability of water/seltzer in high-poverty areas than in low-poverty areas is concerning given high rates of obesity and diabetes in high-poverty areas. As morbidity and mortality associated with low-calorie–related diseases continue to impact individuals, families, communities, and institutions, public health experts must also continue to explore opportunities to address not only the social, but also commercial, determinants that maintain conditions for poor health.

## Abbreviations Used

CT: census tract
CVS: retail pharmacy chain
FPL: Federal Poverty Level
NEMS-B: Nutrition Environment Measures Survey for Beverages
NYC: New York City
NYS: New York State
SD: standard deviation
SNAP: Supplemental Nutrition Assistance Program
